# Long-Term Efficacy of Inferior Oblique Myectomy Accompanied with Tenon’s Capsule Closure: Objective Analysis Using Nine-Gaze Photographs

**DOI:** 10.3390/bioengineering10030352

**Published:** 2023-03-12

**Authors:** Chang Ki Yoon, Hee Kyung Yang, Sang Beom Han, Jeong-Min Hwang

**Affiliations:** 1 Department of Ophthalmology, Seoul National University College of Medicine, Seoul National University Hospital, Seoul 03080, Republic of Korea; 2 Department of Ophthalmology, Seoul National University College of Medicine, Seoul National University Bundang Hospital, Seongnam 13620, Republic of Korea; 3 Department of Ophthalmology, Kangwon National University College of Medicine, Kangwon National University Hospital, Chuncheon 24289, Republic of Korea

**Keywords:** IO myectomy, Tenon’s capsule, IO overaction, SO underaction, computerized analysis

## Abstract

**Background:** The aim is to evaluate the long-term efficacy of inferior oblique (IO) myectomy combined with Tenon’s capsule closure to prevent muscle reattachment to the sclera. **Methods:** We retrospectively reviewed the medical records of 18 patients with primary and secondary IO overaction who underwent IO myectomy accompanied by Tenon’s capsule closure. Patients were followed up for at least 1 year after the surgery. The main outcome measures included oblique muscle dysfunction, which was objectively graded through computerized analysis of nine-gaze photographs, and the amount of vertical deviation in the primary position using alternate prism cover testing. **Results:** After a mean follow up of 2.5 years, the grade of IO overaction decreased from +2.2 ± 1.0 to −0.8 ± 1.0 (*p* < 0.001). In patients with secondary IO overaction with superior oblique (SO) palsy, SO underaction improved from −2.2 ± 1.5 to −0.2 ± 1.8 (*p* = 0.006). Successful vertical deviation in the primary position of seven prism diopters or less was achieved in 83.3% of the patients. Underaction of the IO was observed in 11.1% of patients, whereas none of the patients showed antielevation syndrome. **Conclusion**: IO myectomy combined with Tenon’s capsule closure might be safe and effective for the treatment of primary and secondary IO overaction in the long term.

## 1. Introduction

Inferior oblique (IO) muscle overaction manifests when the gaze is directed to the field of action of the IO, showing an overelevation of the eye during adduction. There are two types of IO overaction––primary and secondary. The etiology of primary IO overaction is unclear and is frequently accompanied by horizontal strabismus, while secondary IO overaction is usually associated with ipsilateral superior oblique (SO) muscle palsy.

For the treatment of primary and secondary IO overaction, several weakening procedures performed on the IO muscle were introduced, including recession, myotomy, myectomy, anterior transposition, and disinsertion [1,2,3,4,5]. Among these methods, IO myectomy has gained popularity because of its relatively simple surgical procedure and its self-grading contribution, in which the surgical effect strongly correlates with the magnitude of preoperative deviation [6,7]. However, recurrence of IO overaction is frequently encountered over time after IO weakening procedures [8,9]. Meanwhile, IO myectomy can also induce overaction of the SO muscle as well as a weakening of IO function [10].

A modified method of IO myectomy that includes additional closure of the Tenon’s capsule after tucking the remaining muscle stump can be helpful for the prevention of reattachment of the IO muscle to the sclera [11]. However, the efficacy of additional Tenon’s closure after an IO myectomy has not been objectively evaluated in the literature. We have previously developed a method for objective analysis of grading oblique muscle dysfunction using computerized analysis of nine-gaze photographs [12]. In this study, using this computerized objective analysis method,^11^ we evaluated the efficacy of this modified procedure in reducing IO overaction and preventing its recurrence up to at least 1 year after surgery.

## 2. Patients and Methods

The medical records of patients with primary or secondary unilateral IO overaction who underwent an IO myectomy combined with closure of the Tenon’s capsule, under the care of one surgeon (JMH) between January 2001 and January 2007, were retrospectively reviewed. Patients who had digitalized nine-gaze photographs with sufficient resolution for analysis and those who were followed up for at least 1 year after surgery were included in the study. Patients who underwent additional vertical rectus muscle surgery or any vertical transposition of the horizontal rectus muscles were excluded. Finally, 18 patients who underwent an IO myectomy with additional closure of the posterior Tenon’s capsule were included for analysis.

Criteria for the diagnosis of primary IO overaction were as follows: (1) IO overaction, (2) no evidence of ipsilateral superior oblique (SO) underaction, and (3) a negative Bielschowsky head tilt test. Secondary IO overaction associated with SO palsy was defined as follows: (1) IO overaction, (2) ipsilateral SO underaction greater than grade 2, (3) a positive Bielschowsky head tilt test, (4) a larger amount of vertical deviation during ipsilateral tilt compared to contralateral tilt of more than 4 prism diopters (PD), and (5) a subjective excyclotorsion measured by the double Maddox rod test over 3°, or excyclotorsion on fundus photographs taken with an internal fixator [10].

IO myectomy was performed as the method described by McNeer et al. [7]. Briefly, a myectomy of the IO muscle of 8–10 mm between the IO muscle insertion and the temporal border of the inferior rectus was performed via clamping with two hemostats. After removal of the muscle segment, the opening in the posterior Tenon’s capsule was repaired with one or two bites of 6-0 polyglactin 910 sutures (Coated Vicryl^®^, Ethicon, UK).

Oblique muscle dysfunction was graded based on the measured angular difference between the adducting eye and the contralateral abducting eye during elevation for IO and depression for SO. The rotating angle along the Listing plane was measured by one of the authors (CKY) using computerized analysis of nine-gaze photographs.^12^ Grading of the oblique muscle dysfunction was performed on a scale of −4 to +4, based on the angular differences measured using the computerized analysis, as follows: grade −4 = angular distance of −20°; grade −3 = angular distance of −15°; grade −2 = angular distance of −10°; grade −1 = angular distance of −5°; grade +1 = angular distance of 5°; grade +2 = angular distance of 10°; grade +3 = angular distance of 15°; and grade +4 = angular distance of 20°.

The amount of vertical deviation in the primary position and the presence of a head tilt of 5 degrees or more were noted before and after surgery. Successful motor alignment was defined as a vertical deviation of 7 prism diopters or less in the primary position.

The main outcome measures were oblique muscle dysfunction graded using the objective computerized analysis and the amount of vertical deviation in the primary position. Preoperative and postoperative values were compared using a Wilcoxon signed-rank test. 

## 3. Results

A total of 5 patients (27.8%) with primary IO overaction and 13 patients (72.2%) with secondary IO overaction associated with SO palsy were included in this study. Patients’ age at surgery ranged from 3 to 21 years, with a mean of 8.1 ± 5.0 years. Nine (50%) patients were female, and the remaining nine (50%) patients were male. The right eye was affected in 6 patients (33.3%), and the left eye was affected in 12 patients (66.7%). Horizontal strabismus surgery was performed simultaneously in four patients (22.2%). The follow-up period ranged from 12 to 53 months, with a mean value of 30.3 ± 14.6 months (Table 1). Abnormal head tilt was present in 13 patients (72.2%), vertical misalignment in 11 patients (61.1%), and 2 patients (11.1%) had intermittent diplopia.

**Figure 1:**
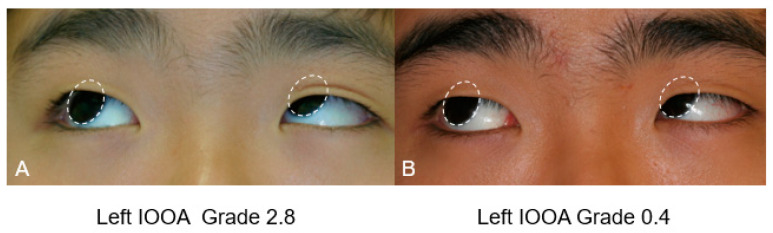
Objective analysis of left inferior oblique overaction (IOOA) in a patient looking at the upper rightward direction before (**A**) and after (**B**) surgery. The corneal contour of both eyes were extrapolated from nine-gaze photographs. The curved line of the corneal margin that is visible from the original photograph was transcribed (dashed line) and a full ellipse was extrapolated so the corneal contour can be drawn for photographic analysis [12].

The mean grade of preoperative IO overaction in all patients was +2.2 ± 1.0, which decreased to −0.8 ± 1.0 (*p* < 0.001). Postoperative IO overaction of grade +1 remained in only one eye (5.5%). In the 13 patients with secondary IO overaction due to SO underaction, the mean grade of preoperative SO underaction was −2.2 ± 1.5, which improved to −0.2 ± 1.8 at the last follow-up examination (*p* = 0.006) (Table 1). A representative figure of oblique muscle dysfunction measured by computerized analysis of nine-gaze photographs is shown in Figure 1. In the contralateral eye, there was no statistically significant change observed in the mean grade of oblique dysfunction after surgery. However, eight patients (44.4%) developed contralateral IO overaction of grade +1 or more, which was not observed before the surgery in any of the patients. (Table 2)

The mean angle of preoperative vertical deviation in the primary position was 15.3 ± 10.2 PD. At the last follow-up examination, vertical deviation in the primary position significantly reduced to a mean value of 2.3 ± 5.6 PD (*p* = 0.002). Fifteen patients (83.3%) showed a vertical deviation of 7 PD or less at the last follow-up examination. Three patients required additional surgery of the vertical rectus muscles. Fifteen patients (83.3%) showed no significant head tilt at the last follow-up examination, while three patients (16.7%) had persistent head tilt.

Two patients (11.1%) with SO palsy showed moderate IO underaction of grade −2 and −3 after surgery. The degree of preoperative IO overaction in these patients was relatively mild as grade 1 and 2, and the amount of hypertropia was 4 PD and 6 PD. Surgery had been performed for persistent head tilt that was corrected after surgery, and postoperative IO underaction did not produce any vertical deviation in the primary position. No patient showed antielevation syndrome.

## 4. Discussion

In this study, an IO myectomy combined with posterior Tenon’s capsule repair showed good long-term results after a mean follow up of 2.5 years. The strength of our study is that we evaluated the long-term effect of an IO myectomy combined with posterior Tenon’s capsule repair using an objective computerized method to quantify ocular oblique muscle dysfunction, which supports the validity and reliability of our results. The grade of IO overaction significantly decreased from +2.2 ± 1.0 to −0.8 ± 1.0, and only one patient (5.5%) showed persistent IO overaction of grade +1. Successful vertical deviation in the primary position was obtained in 83.3% of the patients, and no patient showed antielevation syndrome.

The grading of oblique muscle dysfunction and indications for surgery varies widely among previous reports [8,13,14,15,16,17]. In addition, objective methods to quantify ocular oblique muscle dysfunction have rarely been attempted [12]. Toosi and von Orden [15] reported a 10.3% incidence of persistent postoperative IO overaction and no IO underaction after an IO myectomy. They used a deviometer and an alternate prism cover test to measure oblique muscle function and vertical deviation, but the grading of oblique muscle dysfunction was not specifically described [15]. Parks [8] noted a 37% incidence of persistent postoperative IO overaction and an 8% incidence of IO underaction after a myectomy in 86 eyes at the insertion site. Parks [8] defined any degree of overelevation in adduction as postoperative persistent overaction and did not provide any clear description about the grading of oblique muscle dysfunction. Davis et al. [13] noted a 5% incidence of postoperative overaction and a 3% incidence of underaction after an IO myectomy. They considered 10 PD difference in the adducted and elevation position as IO overaction grade 1. Our results are comparable to Davis’ results in terms of persistent postoperative residual IO overaction (5.5%); however, postoperative IO underaction was slightly more frequent in our study (11.1%).

Successful reduction of vertical deviation in the primary position to 7 PD or less was found in 83.3% in our study, with a mean decrease of 13 PD, which is comparable with previous reports [10,13,15,16]. Simons et al. [10] reported that 80% of patients with SO palsy had a final vertical deviation of 7 PD or less in the primary position after an IO myectomy or recession. Toosi and von Norden^15^ reported a mean vertical change of 11 PD in the primary position after an IO myectomy. Shipman and Burke [16] reported a 14 PD reduction of hyperdeviation in the primary position, and Davis et al. [13] reported 8 PD reduction after a unilateral IO myectomy.

There were no significant complications, such as adherence syndrome, and as was suspected, no antielevation syndrome was found [8,18]. Parks described hypotropia developing after IO weakening, of which the restriction in elevation is greater in abduction than it is in adduction [8]. Stein and Ellis [18] suggested that unilateral anterior transposition or 10 mm recession of the IO muscle may promote limitations in elevation and abduction in the operated eye. Mims and Wood [19] asserted that this antielevation syndrome can be treated with a nasal IO myectomy. The antielevation described by Stein and Ellis [18] did not occur after a myectomy, because a myectomy does not displace the ancillary origin of the IO anteriorly [13,20].

In the present study, 44.4% of patients developed contralateral IO overaction of grade +1 or more after unilateral IO myectomy. There have been many reports concerning contralateral IO overaction after unilateral IO weakening [14,15,16,17,18]. Raab and Costenbader [14] noted that IO overaction developed one third of the time in the contralateral eye after a unilateral myectomy when the second eye had a normal IO function. It developed more than two thirds of the time when the second eye had slight IO overaction [14]. Fleming [21] stated that ipsilateral SO action is unopposed with surgical weakening of the IO muscle, causing incyclotorsion of the operated eye and subsequent contralateral IO muscle overaction to compensate for this incyclotorsion.

There are several limitations in this study. First, a relatively small number of patients were included and the results of the modified IO myectomy procedure were not compared to a control group. Further prospective studies, including a large patient group for comparison between IO myectomy combined with Tenon’s capsule closure and a simple IO myectomy, are therefore warranted to gain a better understanding of the efficacy of this modified procedure. Second, nine-gaze photographs may not show the true amount of oblique muscle dysfunction, and subtle underaction may not be fully revealed until the patient tries hard to reach an extreme gaze [12]. For this reason, the examiner used a target for fixation at 50 cm distance from the patient to make the patient look as far as possible at all gaze positions. A more standardized method for taking photographs would be better for experimental purposes. However, we believe that this objective analysis can improve the accuracy and reproducibility of quantitative assessment of oblique muscle dysfunction.

In conclusion, an IO myectomy combined with posterior Tenon’s capsule repair is safe and effective in treating primary and secondary IO overaction in the long-term with no significant complications.

## Figures and Tables

**Table 1:** Demographics of patients.

Age (years)	8.1 ± 5.0
Male (n)	9 (50%)
Primary IOOA (n)	5 (27.8%)
Secondary IOOA with SO palsy (n)	13 (72.2%)
Right eye (n)	6 (33.3%)
Left eye (n)	12 (66.7%)
Horizontal strabismus surgery (n)	4 (22.2%)
Follow-up period (months)	30.3 ± 14.6

n, number of patients; IO, inferior oblique muscle; OA, overaction; SO, superior oblique muscle.

**Table 2:** Oblique muscle dysfunction before and after inferior oblique myectomy accompanied with Tenon’s capsule closure in the operated eye and contralateral eye.

Muscle	Preoperative	Postoperative	*p* Value *
Ipsilateral IO	+2.2 ± 1.0	−0.8 ± 1.0	<0.001
Ipsilateral SO	−2.2 ± 1.5	−0.2 ± 1.8	0.006
Contralateral IO	−0.1 ± 1.3	+0.0 ± 1.3	0.726
Contralateral SO	+0.2 ± 1.7	+0.3 ± 1.3	0.761

IO, inferior oblique muscle; SO, superior oblique muscle. * Wilcoxon signed-rank test.

## Data Availability

Not applicable.
